# Increasing work-place healthiness with the probiotic *Lactobacillus reuteri*: A randomised, double-blind placebo-controlled study

**DOI:** 10.1186/1476-069X-4-25

**Published:** 2005-11-07

**Authors:** Py Tubelius, Vlaicu Stan, Anders Zachrisson

**Affiliations:** 1 Tetra Pak Occupational Health and Safety AB, Ruben Rausings Gata, 221 86 Lund, Sweden; 2 BioGaia AB, St Lars Väg 42A, 220 09, Lund, Sweden

## Abstract

**Background:**

Short term illnesses, usually caused by respiratory or gastrointestinal diseases are disruptive to productivity and there is relatively little focus on preventative measures. This study examined the effect of the probiotic *Lactobacillus reuteri *protectis (ATCC55730) on its ability to improve work-place healthiness by reducing short term sick-leave caused by respiratory or gastrointestinal infections.

**Methods:**

262 employees at TetraPak in Sweden (day-workers and three-shift-workers) that were healthy at study start were randomised in a double-blind fashion to receive either a daily dose of 10^8 ^Colony Forming Units of *L. reuteri *or placebo for 80 days. The study products were administered with a drinking straw. 181 subjects complied with the study protocol, 94 were randomised to receive *L. reuteri *and 87 received placebo.

**Results:**

In the placebo group 26.4% reported sick-leave for the defined causes during the study as compared with 10.6% in the *L. reuteri *group (p < 0.01). The frequency of sick-days was 0.9% in the placebo group and 0.4% in the *L. reuteri *group (p < 0.01). Among the 53 shift-workers, 33% in the placebo group reported sick during the study period as compared with none in the *L. reuteri *group(p < 0.005).

## Introduction

The general well-being in work-places is receiving increased attention in Sweden. Not only is the well-being and healthiness important to the individual himself, but also important to fellow co-workers, family members and last but not least to the success of the company. During the last few decades increased focus have been on how to increase well-being by offering company-sponsored health care, memberships in fitness centres and similar programs aiming to prevent and cure disease and to increase physical activity and awareness on health issues.

Comparatively little attention has been on the diet and how a daily healthy diet impacts on general health with the exception of anti-obesity initiatives.

The cost due to short term sick-leave is in Sweden alone estimated to more than 2.2 billion € [1] and a majority (50–60%) of the episodes are caused by diseases in the respiratory tract (common cold) and gastrointestinal infections [2].

Of special interest is the well-being among shift-workers as this group is known to be at significantly higher risk to attract short-term illnesses such as the common cold and gastroenteritis [3].

Probiotics, i.e. naturally occurring bacteria with health benefits are gaining wider acceptance. These bacteria, commonly from the *Lactobacillus *family have been demonstrated to have numerous potentially important benefits in terms of gut health and immunity [4], but very few studies address how these effects translate into health benefits in normal populations.

Recently, it was demonstrated in a double blind study [5] that gastrointestinal illnesses and febrile episodes could be significantly reduced in babies attending day-care centers. This was achieved by adding the probiotic *Lactobacillus reuteri *to infant formula during a 12 week long study period.

This study aimed to evaluate if similar effects could be achieved in adults and if the addition of the probiotic *L. reuteri *as a daily dietary supplement can improve work-place healthiness.

## Materials and methods

Healthy volunteers were recruited among employees at Tetra Pak in Sweden. The criteria for participation were that they should be symptom-free at Day 0, between 18–65 years of age, willing to comply with the protocol and that signed informed consent was obtained. The study protocol was approved by the ethical committee at Lund University Hospital.

The overall study period was set to 80 days. The subjects were allowed to miss study treatment occasionally, but not for more than a total of 7 days.

The volunteers were randomised in a double-blind fashion to take a daily probiotic drinking straw together with at least 100 ml liquid. Each straw delivered 10^8 ^Colony Forming Units (CFU) of *L. reuteri *protectis, ATCC55730 or placebo.

During the study period the volunteers were asked to report in a diary format any illness symptoms related to the respiratory tract and/or the gastrointestinal tract resulting in sick-leave, and if so, the duration of the sick-leave. They were also asked to report if they had taken the study product as instructed or, if applicable, how many days they missed taking the study product. The diaries were distributed on Day 0 together with the study product and were collected after finalisation of the study period.

Subjects were randomly assigned to L Reuteri (50%), Placebo (50%) in blocks of 4 by a person handing out a sequentially numbered package, containing either L Reuteri or Placebo, together with a diary marked with the same randomisation number. The packages containing either L Reuteri or Placebo were identical in appearance. The randomisation list was generated bythe data management company and used for sorting and numbering the packages by another person. A list in a sealed envelope was kept by the sponsor. Randomisation envelopes were also generated bythe data management company, and kept for safety by the sponsor. The list of randomisation was kept confidential by the statisticians until all results had been generated and sealed.

## Results

After ethical committee approval had been granted, informed consent was obtained from 262 employees who started treatment. 132 were randomised to receive *L. reuteri *and 130 randomised to placebo. 38 subjects in the *L. reuteri *group and 43 in the placebo group failed to comply with the full protocol requirements and were withdrawn from further analysis. In all cases the reason for non-compliance was that they failed to take the study treatment in accordance with the protocol.

The demographic data for the remaining 181 subjects are given in table 1. There were no statistically significant demographic differences between the two groups.

**Table 1:** Demographic data

	***L. reuteri *Group n = 94**	**Placebo Group n = 87**
Mean age (years)	44	44
Male / Female (%)	65/35	71/29
Shift work (%)	28	31

**Figure 1:**
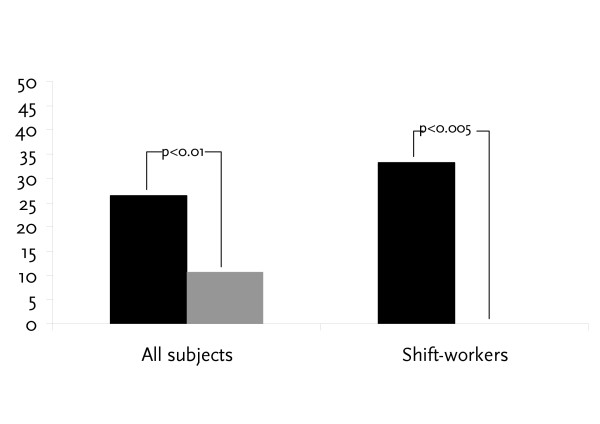
Proportion (%) of subjects reporting sick during the study. ■ = Placebo;  = *L. reuteri.*

The data for symptoms and sick-leave are given in table 2 and figure 1. In the placebo group 23 of 87 subjects reported sick-leave during the study. The corresponding number in the *L. reuteri *group was 10 of 94 (p < 0.01). Consequently, the percentage of sick-days of working days fell from 0.9% in the placebo group to 0.4% in the *L. reuteri *group. This difference was also statistically significant (p < 0.01). However, the median length of sick-leave among the subjects who reported any sick-leave was equal in the two groups, 3 days.

**Table 2:** Sick-leave.

	*L. reuteri ***Group n = 94**	**Placebo Group n = 87**	**Significance**
No. of subjects reporting sick-days (%)	10 (11)	23 (26)	p < 0.01, Pearsons χ^2^
number of sick-days for individuals reporting sick-leave, median	3	3	n.s.
Frequency of sick days*, whole groups	0.4%	0.9%	p < 0.01, Kruskal-Wallis

* Frequency of sick days = (reported number of sick days/total number of study days) × 100

Among the 53 shift-workers in the study, 9 of 27 (33%) in the placebo group reported sick-leave as compared with none of the 26 (0%) in the *L. reuteri *group (p < 0.005, Fisher's exact) (figure 1).

There were no adverse events reported during the study.

## Discussion

The proportion of subjects that were withdrawn for reasons of non-compliance was fairly high, 31%. This can most probably be explained by the study design itself in combination with the fairly long study duration. When the study was designed it was decided that the study staff should meet the study subjects as little as possible in order to minimise any placebo effect as it was assumed that frequent contacts could increase the individual subject's awareness of health issues beyond the normal behaviour.

The outcome of the study demonstrates that daily intake of *L. reuteri *can reduce the proportion of subjects reporting sick from gastrointestinal or respiratory tract diseases by 60%. The effect was highly statistically significant and similar to the findings by Weizman et al [5], where small children in day-care centres had a 70% lower frequency of absence when given *L. reuteri *as compared with placebo.

As demonstrated elsewhere [6-8], *L. reuteri *is efficient both in preventing and treating acute diarrhoea and gastroenteritis in young children. In a study on healthy adults it was shown that *L. reuteri *was able to stimulate the immune system by recruiting CD4+-cells [9]. Such stimulation by *L. reuteri *has been observed in animal models and is associated with an improved response to pathogen infection [10]. Although the exact mechanism of action cannot be defined from our study it is likely that such an immune-stimulation lies behind the reduced morbidity in the subjects taking *L. reuteri*. This stimulation may also explain why the beneficial effect of *L. reuteri *in our study was specifically apparent among shift-workers. This subset consisted of 31% of the total study groups and therefore some caution is warranted when interpreting this result. Nevertheless, shift-workers are known to be at risk for having a weaker immuno-defence as compared to those working day-time shifts only [3]. Consequently it can be argued that shift-workers would benefit relatively more by the immuno-stimulating effect of *L. reuteri*.

In conclusion, the present study demonstrates that *L. reuteri *is effective to promote work-place healthiness. In the studied population sick-days caused by respiratory or gastrointestinal diseases could be reduced by 55% by the use of *L. reuteri *group as compared with the placebo group. Translated to the total Swedish work-force, this translates to a total of 4.3 million working days of improved productivity per year (3.9 million employed, 220 working days per year and 0,5% "saved" days). Our results indicate that the effect on shift-work productivity could probably be even more profound but this issue should be addressed in further studies.

## List of abbreviations used

CFU = Colony Forming Units

## Competing interests

PT and VS declare that they have no competing interests. AZ is a stockholder and an employee of Biogaia AB that holds several patents on *Lactobacillus reuteri.*

## Authors' contributions

PT and VS participated in the design of the study and were responsible for the execution of the study. AZ conceived the study and drafted the design and the manuscript. All authors read and approved the final manuscript.
